# Septic arthritis following intra-articular corticosteroid injections: a retrospective analysis

**DOI:** 10.1007/s00402-026-06262-y

**Published:** 2026-03-09

**Authors:** Marlena Rose Mueller, Travis Cleland, Corrilynn O. Hileman, Andrew Olsen, Robert Wissner, Kimberley R. Monden

**Affiliations:** 1 University of Minnesota Medical School, MN, Minneapolis, USA; 2 Crystal Clinic Orthopedic Center, OH, Akron, USA; 3 The MetroHealth System, Case Western Reserve University School of Medicine, OH, Cleveland, USA; 4 Summa Health, OH, Cleveland, USA; 5 The Spine and Pain Institute, OH, Akron, United States of America

**Keywords:** Septic arthritis, Intra-articular injections, Procedural complications, Corticosteroids

## Abstract

**Introduction:**

Septic arthritis is a rare but devastating complication of intra-articular corticosteroid injection (CSI), associated with significant medical morbidity and poor clinical outcomes. Although previous studies have examined risks associated with CSI, few have tracked patients long-term. This study evaluates the incidence, timing, and patient characteristics related to iatrogenic septic arthritis within 6 months of receiving a large joint CSI, offering new insights to inform clinical practice.

**Materials and methods:**

A retrospective, descriptive cohort study was conducted using SlicerDicer, a software stratification system within Epic, to identify patients diagnosed with septic arthritis within six months of receiving an intra-articular CSI of the hip, knee, or shoulder. Data were collected from a single institution over a 10-year period (July 1, 2010 to July 1, 2020). Individual chart review was used to obtain patient demographics, clinical characteristics, and procedural details for identified cases.

**Results:**

Of 15,021 intra-articular corticosteroid injections performed, 14 cases of septic arthritis were identified within 6 months of the procedure, resulting in an incidence rate of 0.093%. Of the affected patients, 21% had underlying inflammatory arthritis, and 21% had underlying comorbidities resulting in immunosuppression. The median time to diagnosis was 3.5 (range 1–16) weeks post-injection, with a bimodal distribution of infections occurring primarily between 1 and 3 and 7–10 weeks post-injection. *Staphylococcus aureus* (42%) and coagulase-negative *Staphylococcus* species (36%) were the most commonly isolated organisms.

**Conclusions:**

Large joint intra-articular CSI presents a low risk (< 0.1%) for developing septic arthritis, but can present up to 16 weeks post-injection. The preliminary observation of bimodal timing and delayed presentation of septic arthritis suggests that a standard two-week surveillance window may not be long enough to fully capture all infections, particularly those involving lower-virulence organisms. These findings highlight the need for informed risk stratification and prolonged vigilance well beyond the immediate post-injection period to identify infectious complications after large joint CSI.

## Introduction

Intra-articular corticosteroid injections (CSI) are frequently used in clinical practice for the management of osteoarthritis and have been shown to provide at least short-term improvements in joint pain and function [1–3]. Despite their widespread use and overall favorable safety profile, intra-articular CSI carries the risk of septic arthritis as a potentially severe complication. This can lead to rapid joint destruction, bacteremia, and sepsis if not promptly diagnosed and treated [4–6]. Septic arthritis represents an orthopedic emergency due to potentially serious and life-threatening outcomes, with an estimated mortality rate between 10 and 15% [7]. Beyond these acute risks, this condition can lead to irreversible joint damage necessitating complex salvage procedures or two-stage joint arthroplasty. In the hip, research indicates that up to 33% of patients experience treatment failure, and some may never progress to a definitive joint replacement following iatrogenic intra-articular infection [8].

Current literature on septic arthritis following intra-articular CSI has several limitations, particularly in terms of the duration of follow-up [9]. Existing studies primarily track clinical outcomes for no more than two weeks post-injection, potentially leading to underreporting of delayed infectious complications [9, 10]. This underscores the need for greater scrutiny of infection timing and the implementation of more extended monitoring periods. The reported incidence of septic arthritis following joint injections is highly variable across studies, ranging from 2 to 80 cases per 100,000 injections [5, 6, 10]. This wide range is largely due to heterogeneous study designs that have variable follow-up periods and include data from multiple injection types (e.g., corticosteroids, hyaluronic acid, and plain arthrocentesis) and multiple joint sizes, making the specific incidence of septic arthritis after large joint CSI more challenging to quantify [6, 11]. Despite this variability, the clinical significance of this complication is substantial, as the incidence in many reports surpasses other serious orthopedic infections such as fracture-related infections, which occur in 10.7 cases per 100,000 persons annually [12].

The purpose of this study was to address critical gaps in the literature by investigating the incidence and timing of septic arthritis after large joint CSI, utilizing an extended clinical observation period of 6 months and excluding non-corticosteroid injections. We hypothesized that the observed frequency of septic arthritis following CSI may be higher than currently reported, in part due to identification of delayed presentations that fall outside traditional monitoring periods and the inherent risk profile associated with corticosteroid use.

## Materials and methods

This single-hospital system retrospective, descriptive analysis was conducted at an academic institution in Cleveland, Ohio, USA, using the Epic Systems Corporation electronic medical record (EpicCare EMR, Verona, WI) for data collection. Following Institutional Review Board approval, we used SlicerDicer, a software stratification system within Epic, to identify all patients over the age of 18 within our institution who received a large joint CSI from July 2010 to July 2020. Per institutional protocol, intra-articular injections were routinely administered in combination with a local anesthetic (typically lidocaine or ropivacaine). Using ICD-9 and ICD-10 codes, we identified patients who were diagnosed with septic arthritis within 6 months of receiving their CSI.

All such cases underwent a manual retrospective chart review (MM, AO, RW) to confirm the diagnosis of septic arthritis and ensure it followed an intra-articular CSI of the same joint. Patients who underwent joint replacement between the time of injection and diagnosis of septic arthritis were excluded from the final sample, as these were found to represent post-operative complications. This extended follow-up period was selected to ensure the capture of insidious, slow-growing organisms such as mycobacteria, which often manifest symptoms significantly later than highly virulent organisms. Additionally, since corticosteroids can mask inflammatory symptoms typically associated with early infection, a 6-month window was used to avoid the premature exclusion of cases with delayed clinical presentation.

Cases of septic arthritis were confirmed using Newman’s criteria: a positive synovial fluid culture, or a clinical diagnosis of septic arthritis with positive blood cultures and a synovial WBC count of > 50,000 cells/mm³ [13]. Chart review was used to collect demographic information of affected patients, including sex at birth, age, and body mass index (BMI). Synovial fluid cultures were also manually reviewed for every patient, including evaluation of cell count, Gram stain, and culture results. Timing between CSI and the diagnosis of septic arthritis was examined and documented for each patient, as well as any surgical interventions required. Other patient data collected included cataloging the joint affected as well as any medical history of diabetes, immunosuppression, tobacco use, inflammatory arthritis, the number of previous corticosteroid injections in the affected joint, and history of recent tooth extraction. Descriptive statistical analyses were used to analyze patient demographics, clinical characteristics, and timing of septic arthritis diagnosis.

## Results

A total of 15,021 patients received an intra-articular CSI of the hip, knee, or shoulder over the designated 10-year period from July 2010–2020. Out of these, 16 patients were diagnosed with septic arthritis of the same joint within 6 months. Two patients were excluded from the results, as they underwent a total knee arthroplasty between the time of their corticosteroid injection and subsequent diagnosis of septic arthritis, representing post-operative infections. The 14 remaining cases found to have a direct association with previous CSI showed an overall incidence of 0.093% or 9.3 cases of septic arthritis per 10,000 large joint CSI.

In total, 14 patients with confirmed septic arthritis were identified following large joint CSI over the 10-year study period. The mean age of affected patients was 61.9 (range 33–89) years, and 50% were female. The mean BMI was 30.9 (range 20–42), and most had underlying medical comorbidities or previously cited risk factors. Three patients had known inflammatory arthritis, three were immunosuppressed, and four were diabetic. Two patients had a tooth extraction between the time of their CSI and the subsequent diagnosis of septic arthritis. Nine patients had a history of a previous corticosteroid injection in the same joint, seven of whom had more than one prior injection. Clinical characteristics of the cases are shown in Table 1.

**Table 1:** Characteristics of patients with septic arthritis

Age	Gender	BMI	Joint	Organism	Patient Characteristics
61	F	38.2	Shoulder	*S. aureus*†	Diabetic
66	M	29.8	Knee	*S . aureus *(blood)†	Immunosuppressed
89	F	27.5	Knee	*S. lugdunensis*	Diabetic
86	F	26.1	Knee	*Staphylococcus *spp.	Diabetic
68	M	30.1	Knee	*S. capitis*	-
70	F	32.1	Knee	*S. aureus*†	Inflammatory arthritis, immunosuppressed
49	F	42.7	Knee	*S. epidermidis*	Smoker
59	M	35.4	Knee	*S. mitis*,* S. caprae*	-
33	M	20.7	Knee	*S. aureus*†	Smoker, immunosuppressed
61	F	38.6	Knee	*S. aureus*†	Diabetic
51	M	29	Hip	*Aggregatibacter*	S/p tooth extraction, smoker, inflammatory arthritis
57	F	23.1	Hip	*S. epidermidis*	Inflammatory arthritis
75	M	26.3	Hip	*S. epidermidis*	S/p tooth extraction
41	M	32.8	Hip	*S. aureus*‡	Smoker

†Methicillin-sensitive *Staphylococcus aureus*

‡Methicillin-resistant *Staphylococcus aureus*

The median time to diagnosis of septic arthritis was 3.5 weeks after injection, with time to diagnosis ranging from 1 to 16 weeks. In this cohort, the distribution of time to diagnosis followed a bimodal pattern, with peaks in infection occurring between 1 and 3 and 7–10 weeks post-injection (range 1–16 weeks). The knee was the most commonly affected joint (*n* = 9), and the most frequently isolated organism was *Staphylococcus aureus* (*n* = 6).

Regarding clinical outcomes and management, all 14 patients required hospitalization for intravenous antibiotic administration. One patient, who was immunosuppressed due to active chemotherapy, did not undergo surgical intervention due to the initial presentation of septic shock, which led to rapid medical decompensation. This patient ultimately died from complications related to septic arthritis after an extended hospital course, leading to an overall mortality rate of 7.14% for patients with septic arthritis following large joint CSI in this cohort.

The remaining 13 patients underwent initial surgical debridement of their involved joint, 10 of whom (71.4%) had successful clearance of their infection after a single debridement and IV antibiotics alone. Five patients (35.7%) ultimately required salvage procedures or definitive joint replacement. One patient with septic arthritis of the hip underwent a Girdlestone procedure at the time of initial incision and drainage, followed by a complex salvage course requiring secondary placement of an antibiotic spacer, with a subsequent third revision for spacer dislocation, and a later antibiotic spacer exchange. At the conclusion of the study period, this patient had not progressed to definitive joint replacement. Four patients (28.5%) successfully cleared their infection, but ultimately required a total joint arthroplasty for destructive arthritis.

Five patients were initially diagnosed with septic arthritis 8 weeks or longer after their CSI. Of these patients, three of the four who survived (75%) required an eventual joint replacement or salvage procedures. In comparison, only one patient (14.3%) out of seven diagnosed with septic arthritis within the first three weeks ultimately required a joint replacement.

## Discussion

Our study found an incidence of 9.3 cases of septic arthritis per 10,000 intra-articular CSI. This value is slightly higher than contemporary estimates reported in the literature. A 2019 nationwide study in Denmark reported an incidence of 8 cases of septic arthritis per 10,000 CSI within a two-week follow-up period [10]. Similarly, an Icelandic study identified 3.7 cases of septic arthritis per 10,000 intra-articular injections of all types [6]. Commonly cited statistics in broader literature reviews place the incidence of septic arthritis following joint injections between 1 in 3,000 and 1 in 50,000, reflecting high variability based on study design and follow-up duration [6, 7, 9, 10, 14]. These rates are much higher than the incidence of native joint septic arthritis, which occurs in approximately 2 cases per 100,000 persons per year [14, 15]. The higher incidence in our cohort likely reflects our specific focus on corticosteroid injections and our extended six-month surveillance period.

A significant finding from this study is the observed frequency of delayed infection onset, with a median time to diagnosis of 3.5 weeks, and some cases presenting as late as 16 weeks post-injection. This extended window of risk is not well-documented in the literature, as most existing studies have either followed patients for no longer than two weeks or did not specify a clear timeline for symptom onset [6, 9, 10]. Our data demonstrated a bimodal distribution of infections, occurring primarily between 1 and 3 and 7–10 weeks after injection, further suggesting that the true incidence of post-injection septic arthritis may be underestimated in existing literature with shorter monitoring periods. Clinicians should maintain a high index of suspicion for this diagnosis even months after CSI. Recognizing the potential for delayed presentation of septic arthritis may lead to earlier diagnosis and treatment, which is essential for reducing morbidity and improving patient outcomes.

Known risk factors for developing septic arthritis after intra-articular injections include elevated BMI, a history of corticosteroid injection, inflammatory arthritis, diabetes, immunosuppression, advanced age (> 60), and recent dental procedures [5–7, 10, 11, 16]. The patient characteristics observed in our study align closely with these established risks, as 71.5% of our cohort had one or more of these comorbidities. Although our observed incidence of post-injection septic arthritis of 0.093% is slightly higher than existing data, this likely reflects the extended 6-month time period evaluated and the specific exclusion of non-corticosteroid injections. Notably, our finding of the mean time to septic arthritis diagnosis of 3.5 weeks suggests existing studies limiting follow-up periods to two weeks may underreport cases of septic arthritis, explaining the lower rates frequently cited in prior research.

The pathogenesis of septic arthritis following CSI is likely multifactorial, with the most common mechanisms supported by the literature being direct inoculation and hematogenous seeding of the puncture tract by circulating pathogens [7, 17]. In our study, most of the organisms isolated were consistent with normal skin flora, supporting iatrogenic inoculation during their procedure as the route of infection. Up to 20% of skin bacteria are found in the deeper layers of the skin in the pilosebaceous units, which are untouched by topical antiseptics. The risk of inoculation is further compounded by tissue coring, a mechanism by which large-bore needles inadvertently introduce bacteria-containing epidermal plugs directly into the joint space [7]. Other recognized mechanisms of post-procedure infection include the activation of a previously quiescent infection and contamination of the injectate [7, 17]. The importance of the sterile technique has been previously demonstrated after a 2017 investigation into an outbreak of 41 cases of post-injection septic arthritis in a single outpatient clinic, where multiple breaches in infection prevention protocols were discovered, including the repeated use of preservative-free products intended to be used as single-dose vials [18].

In our cohort, *Staphylococcus aureus* (*n* = 6) was the most frequently isolated pathogen. This is consistent with its well-established role as a leading cause of septic arthritis due to its virulence and ability to adhere to synovial tissue [5, 15, 18]. Notably, the second most common organisms isolated in our study were coagulase-negative *Staphylococcus* species (*n* = 5). While these organisms are typically regarded as less virulent than *Staphylococcus aureus*, they can still cause significant morbidity, especially in immunocompromised individuals [5, 15]. Due to their low virulence, these infections can present with a more insidious or gradual onset, potentially leading to a delayed clinical presentation and subsequent underreporting in existing literature [7, 17]. This also helps explain the median time to diagnosis of 3.5 weeks in this study. Ultimately, the presence of these organisms, especially in a cohort in which 71.4% had underlying risk factors, highlights the critical need for prolonged clinical vigilance and prompt and targeted antimicrobial therapy to mitigate permanent joint damage.

We notably excluded two patients who underwent total knee arthroplasty between the time of their injection and the subsequent diagnosis of septic arthritis. While these cases were classified as postoperative complications for the purpose of this analysis, they highlight the need for consideration of the time-dependent association between preoperative CSI and periprosthetic joint infection (PJI). Recent systematic reviews indicate that preoperative intra-articular injections are associated with an increased risk of PJI, particularly when administered within three months of surgery [19, 20]. This increased susceptibility may be due to the ability of corticosteroids to impair local host defenses within a newly implanted joint [7]. The sequelae of infection, whether in a native or prosthetic joint, can lead to devastating outcomes. This is further evidenced by a patient in our study with septic arthritis of the hip who underwent a protracted salvage course following an initial Girdlestone procedure. This patient’s failure to progress to definitive joint replacement highlights the management difficulties inherent to these infections, which often include the need for complex salvage procedures and staged revisions [8].

This study is not without limitations, including its retrospective nature and lack of a control group, which prevents a true assessment of risk factors. The reliance on ICD-9/10 codes for patient identification is also a potential source of error. It also remains possible that cases were potentially undercounted if patients presented to outside institutions for the treatment of subsequent infections. Additionally, as a single-center retrospective study with a small sample size (*n* = 14), the generalizability of our findings is limited. The low number of cases lacks sufficient power to support reliable subgroup inference or multivariable regression analysis, preventing us from identifying true independent risk factors for infection, and caution should be exercised when drawing broad conclusions. However, despite these limitations, we were unable to find a similar study with an equivalent prolonged and defined follow-up period, making our findings a valuable contribution to the literature.

Understanding the incidence, timing, and risk factors for post-CSI septic arthritis is critical for improving patient selection, informed consent discussions, and guidance of appropriate post-injection monitoring protocols. This study’s extended follow-up period and focus on large joints provides valuable insights to help clinicians better assess and manage the risks associated with intra-articular corticosteroid injections. Future studies could build on these findings by incorporating matched cohorts to more accurately identify patient and procedure-specific risk factors.

## Conclusion

The overall incidence of septic arthritis following large joint CSI was low (0.093%), yet our findings support our hypothesis that the true incidence may be slightly higher than previously reported when longer follow-up intervals are considered. Notably, cases of septic arthritis were observed to manifest much later than previously reported, up to 16 weeks post-injection. Our findings suggest that standard two-week surveillance protocols may be insufficient to capture delayed presentations, as nearly half of the cases in this cohort were diagnosed after that window. Despite the overall low incidence of septic arthritis after CSI, our findings of bimodal presentation and late-onset cases highlights the need for prolonged vigilance, especially in high-risk patients. Clinicians should maintain a high index of suspicion for septic arthritis for up to 16 weeks after intra-articular CSI, especially in patient populations with significant medical comorbidities.

## Data Availability

No datasets were generated or analysed during the current study.
